# Nuclei-specific differences in nerve terminal distribution, morphology, and development in mouse visual thalamus

**DOI:** 10.1186/1749-8104-9-16

**Published:** 2014-07-10

**Authors:** Sarah Hammer, Gabriela L Carrillo, Gubbi Govindaiah, Aboozar Monavarfeshani, Joseph S Bircher, Jianmin Su, William Guido, Michael A Fox

**Affiliations:** 1 Virginia Tech Carilion Research Institute, 2 Riverside Circle, Roanoke, VA 24016, USA; 2 Roanoke Valley Governor School, 2104 Grandin Road SW, Roanoke, VA 24015, USA; 3 Department of Psychology, Virginia Tech, 109 Williams Hall, Blacksburg, VA 24061, USA; 4 Department of Biological Sciences, Virginia Tech, 2125 Derring Hall, Blacksburg, VA 24061, USA; 5 Virginia Tech Carilion School of Medicine, 2 Riverside Circle, Roanoke, VA 24016, USA; 6 Department of Anatomical Sciences and Neurobiology, University of Louisville, 511 South Floyd, Louisville, KY 40202, USA

**Keywords:** Retina, Thalamus, Retinogeniculate, Lateral geniculate nucleus, Axon, Retinal terminal, Nerve terminal

## Abstract

**Background:**

Mouse visual thalamus has emerged as a powerful model for understanding the mechanisms underlying neural circuit formation and function. Three distinct nuclei within mouse thalamus receive retinal input, the dorsal lateral geniculate nucleus (dLGN), the ventral lateral geniculate nucleus (vLGN), and the intergeniculate nucleus (IGL). However, in each of these nuclei, retinal inputs are vastly outnumbered by nonretinal inputs that arise from cortical and subcortical sources. Although retinal and nonretinal terminals associated within dLGN circuitry have been well characterized, we know little about nerve terminal organization, distribution and development in other nuclei of mouse visual thalamus.

**Results:**

Immunolabeling specific subsets of synapses with antibodies against vesicle-associated neurotransmitter transporters or neurotransmitter synthesizing enzymes revealed significant differences in the composition, distribution and morphology of nonretinal terminals in dLGN, vLGN and IGL. For example, inhibitory terminals are more densely packed in vLGN, and cortical terminals are more densely distributed in dLGN. Overall, synaptic terminal density appears least dense in IGL. Similar nuclei-specific differences were observed for retinal terminals using immunolabeling, genetic labeling, axonal tracing and serial block face scanning electron microscopy: retinal terminals are smaller, less morphologically complex, and more densely distributed in vLGN than in dLGN. Since glutamatergic terminal size often correlates with synaptic function, we used *in vitro* whole cell recordings and optic tract stimulation in acutely prepared thalamic slices to reveal that excitatory postsynaptic currents (EPSCs) are considerably smaller in vLGN and show distinct responses following paired stimuli. Finally, anterograde labeling of retinal terminals throughout early postnatal development revealed that anatomical differences in retinal nerve terminal structure are not observable as synapses initially formed, but rather developed as retinogeniculate circuits mature.

**Conclusions:**

Taken together, these results reveal nuclei-specific differences in nerve terminal composition, distribution, and morphology in mouse visual thalamus. These results raise intriguing questions about the different functions of these nuclei in processing light-derived information, as well as differences in the mechanisms that underlie their unique, nuclei-specific development.

## Background

The visual thalamus of rodents has served as an important model for exploring the cellular and molecular mechanisms that underlie neural circuit formation. The overwhelming majority of these studies have focused on inputs to and projections from the dorsal lateral geniculate nucleus (dLGN). Relay neurons within dLGN receive strong glutamatergic inputs from retinal ganglion cells (RGCs) and serve as the principle conduit of visual signaling to the cortex. However, relay neurons do not act as passive relays of visual information. The gain of retinogeniculate signal transmission is modulated by nonretinal inputs to dLGN. These nonretinal inputs arise from visual cortex, pretectum, brainstem, thalamic reticular nuclei, and local dLGN interneurons, and they far outnumber the more powerful retinal inputs [1,2]. In fact, nonretinal inputs account for as much as 95% of the nerve terminals in dLGN [1,3-6].

Differences in the functional properties of inputs to dLGN translate into distinct neurochemical and ultrastructural differences in retinal and nonretinal synapses in dLGN. Retinal, cortical, brainstem and inhibitory nerve terminals in dLGN all contain distinct neurotransmitter synthesizing enzymes and synaptic vesicle associated transporter proteins necessary for their specific functions [4,7-12]. At the ultrastructural level, several types of nerve terminals have been described in dLGN, defined by anatomical features such as terminal morphology, synaptic vesicle shape, and mitochondrial appearance [6,13-16]. One set of nerve terminals contain flattened, oval shaped synaptic vesicles, a hallmark feature of inhibitory GABAergic terminals, and are therefore called F terminals [2,14,17]. In dLGN, GABAergic terminals arise from the thalamic reticular nucleus and local inhibitory interneurons [7,18,19]. Other classes of terminals in dLGN contain round (or rather spherical) synaptic vesicles, and these represent excitatory and modulatory inputs from the retina, cortex, brainstem, pretectum and superior colliculus [1]. Glutamatergic retinal terminals are distinguished from all other round-vesicle containing terminals based on their exceptionally large size and pale-colored mitochondria [6,13,14,20]. For this reason retinal terminals in dLGN are termed RLP terminals (for *r*ound synaptic vesicles, *l*arge terminal size, and *p*ale-colored mitochondria). In contrast, nonretinal excitatory/modulatory nerve terminals, which far outnumber RLPs, are termed RSD terminals for their round synaptic vesicles, small terminal size, and dark-colored mitochondria [3,4,6]. In addition to terminal size and mitochondrial appearance, retinal terminals in dLGN are distinguishable from other terminal types as they exhibit complex synaptic arrangements with F terminals, they cluster into complex synaptic zones encapsulated by glial processes (and arrangement termed a glomeruli), and they typically contain invaginations of spine-like structures from either dendrites of dLGN principle neurons or F terminals [2,14,20].

While synaptic organization and morphology in dLGN have been thoroughly explored, other retino-recipient regions of mouse thalamus have received far less attention. Adjacent to the dLGN are the ventral lateral geniculate nucleus (vLGN) and the intergeniculate leaflet (IGL), two thalamic nuclei that receive and process light derived information from the retina. It is important to note that while retinal axons innervate all regions of dLGN and IGL, only the external division of the rodent vLGN receives retinal afferent [21]. For simplicity sake we will be referring to this external division of vLGN throughout this manuscript. In contrast to dLGN, which relays image-forming visual information to the primary visual cortex, vLGN and IGL contribute to functions of the non-image forming accessory visual system, such as irradiance detection, visuomotor responses, and circadian photo-entrainment [22,23]. Source of inputs to vLGN and IGL are similar to that of dLGN, which includes inputs from retina, cortex, superior colliculus, thalamic reticular nucleus, and local interneurons [22,24]. However, it is becoming increasingly clear that different classes of neurons from these nuclei project to distinct nuclei of visual thalamus. For example, while classes of image-forming RGCs, which transmit information regarding contrast, color and motion, project to dLGN, classes of non-image forming RGCs project to vLGN and IGL [23,25-34]. Likewise, distinct classes of cortical neurons innervate dLGN and vLGN. Layer VI cortical neurons project to dLGN, whereas layer V cortical neurons project to vLGN [24,35,36]. In addition to differences in their inputs, projections from relay neurons in dLGN and vLGN widely differ. In contrast to the thalamocortical projections originating from dLGN, projections from vLGN do not enter cortex and instead innervate an array of ipsi- and contralateral structures within hypothalamus, thalamus and midbrain [22].

Despite this knowledge of the circuits associated with the more ventrally located nuclei of rodent visual thalamus, we lack important information regarding the organization, distribution and morphology of nerve terminals in this region. We therefore explored and compared the distribution, morphology and physiology of nerve terminals in vLGN and dLGN using immunolabeling, mouse genetics, anterograde axonal labeling, serial block face scanning electron microscope (SBFSEM) and whole-cell patch recordings. Our results show that nerve terminal composition not only differs between visual thalamic nuclei, but that, retinal terminal development, morphology and physiology also differ significantly in these nuclei.

## Results and discussion

### Distribution of inhibitory and modulatory terminals in mouse visual thalamic nuclei

**Figure 1:**
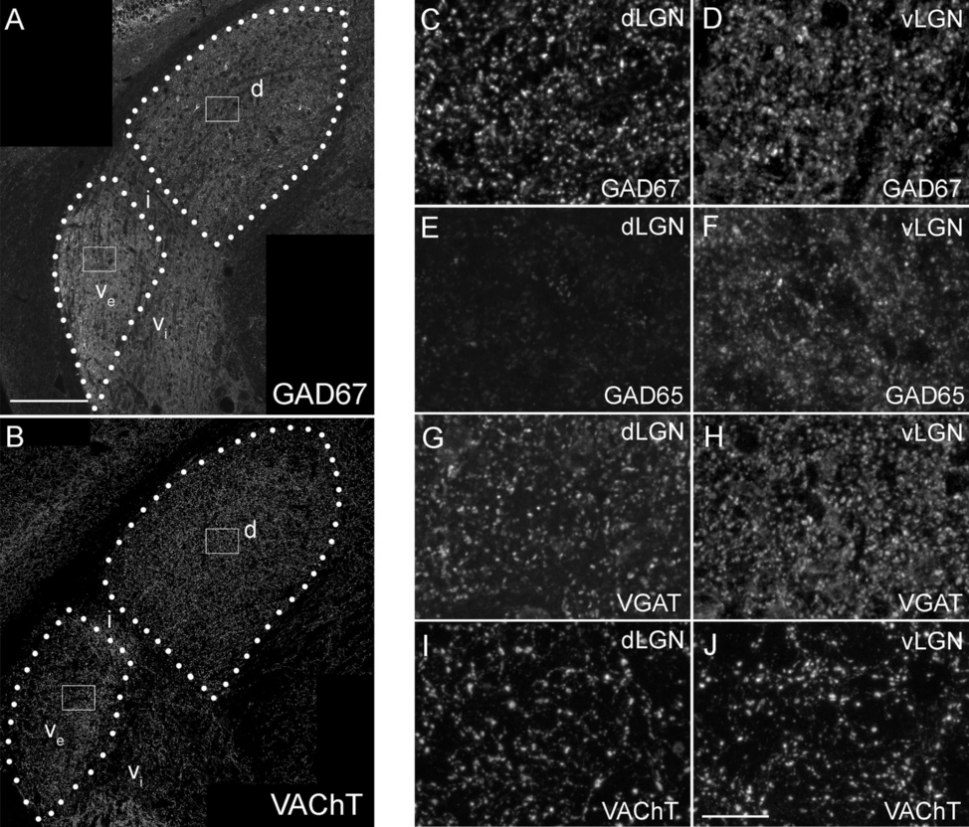
**Distribution of inhibitory and modulatory nerve terminals in subnuclei of mouse visual thalamus. A,B**. Confocal images of immunohistochemistry (IHC) for GAD67 **(A)** and VAChT **(B)** in coronal sections of adult mouse LGN. Outlines of the dorsal lateral geniculate nucleus (dLGN) and the external division of the ventral lateral geniculate nucleus (vLGN) are depicted with white dots. d, dLGN; v_e_, external division of vLGN; v_i_, internal division of vLGN; i, IGL. White boxes depict regions enlarged in **C,D,I,** and **J. C,D**. High magnification images of GAD67-immunoreactivity in dLGN **(C)** and vLGN **(D)** from the regions boxed in **A. E,F**. High magnification images of GAD65-immunoreactivity in dLGN **(E)** and vLGN **(F)**. Note the lack of GAD65-immunoreactivity in dLGN. **G,H**. High magnification images of VGAT-immunoreactivity in dLGN **(G)** and vLGN **(H)**. High magnification images of VAChT-immunoreactivity in dLGN **(I)** and vLGN **(J)** from the regions boxed in **B**. All images are maximum projection confocal images. Scale bar in **A** = 200 μm for **A,B** and in **J** = 25 μm for **C-J**.

To compare the distribution of neurochemically-defined populations of nerve terminals, we performed immunohistochemistry on coronal sections of adult mouse brain. Classes of inhibitory nerve terminals were labeled with antibodies against either cytoplasmic enzymes required to generate GABA (i.e. Glutamate Decarboxylase 65 (GAD65) and GAD67) or synaptic vesicle associated transporters required to fill synaptic vesicles with GABA (i.e. Vesicular GABA Transporter (VGAT)). GAD67-immunoreactivity (IR) was differentially distributed in all three nuclei of mouse visual thalamus, with the highest expression in vLGN and lowest in IGL (Figure 1A). In both vLGN and dLGN (but not in IGL), interneuron cell somata were labeled, and GAD67-positive nerve terminals were observed in both vLGN and dLGN (Figure 1C,D). While the size of individual GAD67-IR terminals appeared similar in dLGN and vLGN, the density of these terminals was considerably higher in vLGN. Terminal density was quantified by measuring the area fraction of each image containing GAD67-IR (in vLGN 22.5% ± 0.6% of images were occupied by GAD67-IR versus 12.3% ± 0.4% in dLGN. Data are shown as mean ± SEM. *P* <0.00001 by Student’s *t*-test).

A more striking difference in the distribution of inhibitory nerve terminals was apparent following GAD65-immunostaining. GAD65 was robustly present in vLGN and IGL but was lowly expressed in dLGN (Figure 1E,F; see [37]). In many brain regions, GAD65 and GAD67 are present in different subsets of inhibitory nerve terminals [38-40]. This appears to be the case in both dLGN and IGL since GAD67^+^/GAD65^-^ terminals were present in dLGN and GAD67^-^/GAD65^+^ terminals were present in IGL (Figure 1A,C-F and [37]). Since terminals in vLGN were labeled with antibodies against both GAD67 and GAD65, it remains unclear whether these terminals co-express both enzymes, or whether these represent two unique populations of inhibitory nerve terminals in vLGN.To confirm differences in inhibitory terminal distribution in dLGN and vLGN, we labeled inhibitory terminals with antibodies against VGAT. These results closely resembled those obtained with GAD67 immunolabeling. VGAT-positive inhibitory terminal size appeared similar in dLGN and vLGN, but the density of these terminals was higher in vLGN (Figure 1G,H).

We next explored the distribution of cholinergic nerve terminals in vLGN and dLGN [4,41]. These modulatory inputs arise from several brainstem nuclei. We immunolabeled these terminals with antibodies directed against Vesicle Associated Choline Transporter (VAChT), which is required to load synaptic vesicles with acetylcholine. VAChT-positive terminals appeared similar in size in vLGN and dLGN (Figure 1B,I,J), and the density of cholinergic terminals in vLGN and dLGN was similar (in vLGN 4.3% ± 0.9% of images were occupied with VAChT-IR versus 5.3% ± 0.5% in dLGN. Data are shown as mean ± SEM. *P* = 0.14 by Student’s *t*-test). Thus, while significant differences were noted in the composition and density of inhibitory terminals in mouse visual thalamic nuclei, the size and density of cholinergic inputs appears to lack nuclei-specific differences.

### Distribution of excitatory nerve terminals in mouse visual thalamic nuclei

**Figure 2:**
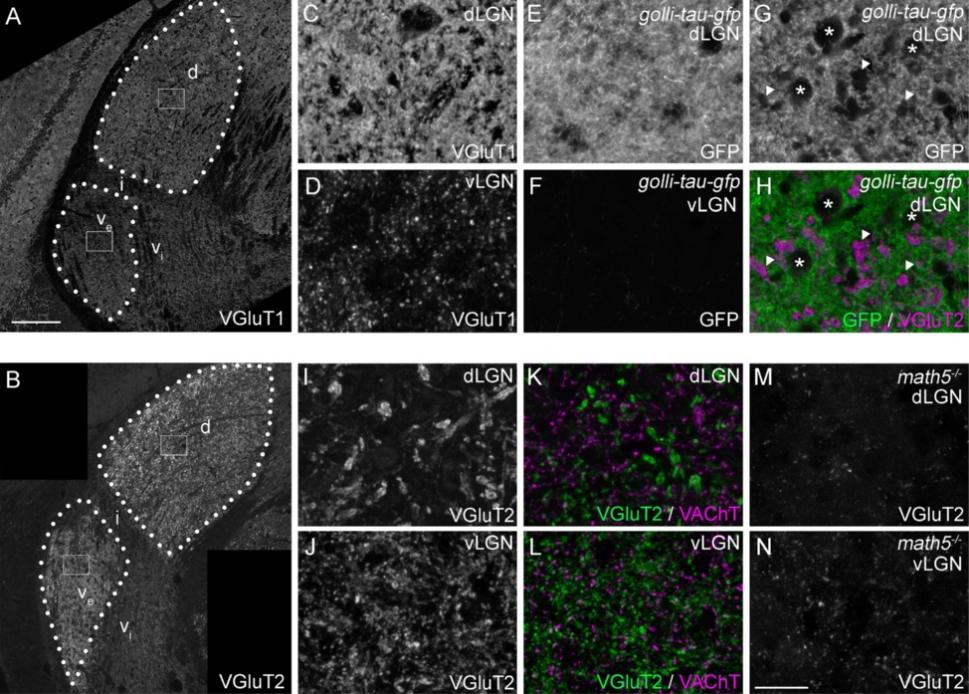
**Distribution of excitatory nerve terminals in subnuclei of mouse visual thalamus. A,B**: Confocal images of immunohistochemistry (IHC) for VGluT1**(A)** and VGluT2 **(B)** in coronal sections of adult mouse lateral geniculate nucleus (LGN). d, dorsal lateral geniculate nucleus (dLGN); v_e_, external division of ventral lateral geniculate nucleus (vLGN); v_i_, internal division of vLGN; i, intergeniculate nucleus (IGL). **C,D**: High magnification images of VGluT1-immunoreactivity in dLGN **(C)** and vLGN **(D)** from the regions boxed in **A**. **E,F**. High magnification images of Green Fluorescent Protein (GFP) distribution in dLGN **(E)** and vLGN **(F)** of adult *Golli-tau-gfp* transgenic mice. GFP was detected by GFP-immunostaining. Layer VI cortical neurons are selectively labeled with tau-GFP in these transgenic mice. **G,H**. A single optical section of a confocal image of GFP **(G,H)**- and VGluT2 **(H)**-immunoreactivity in the dLGN of an adult *Golli-tau-gfp*. GFP-positive cortical axon arbors densely populate the dLGN neuropil. Regions devoid of GFP-immunoreactivity in dLGN are occupied by cell bodies (asterisks), VGluT2-positive terminals (arrowheads) or blood vessels (not labeled here). **I,J**. High magnification images of VGluT2-immunoreactivity in dLGN **(I)** and vLGN **(J)** from the regions boxed in **B**. Note the difference in VGluT2-positive terminal size in dLGN and vLGN. **K,L**. High magnification images of VGluT2 (green) and VAChT (magenta)-containing nerve terminals in dLGN **(K)** and vLGN **(L)**. VGluT2-positive terminals in dLGN are not only larger than those in vLGN, but are dramatically larger than other types of terminals in dLGN. **M**,**N**. To demonstrate that VGluT2-positive terminals originate from retinal ganglion cells, we assessed their distribution in LGN of adult *math5*^*-/-*^ mutants, which lack retinogeniculate projections. Few, if any, nerve terminals appeared to contain VGluT2 in these mutants. All images are maximum projection confocal images except **G,H**. Scale bar in **A** = 200 μm for **A,B** and in **N** = 25 μm for **C-N**.

We next examined glutamatergic inputs in vLGN and dLGN, which arise mainly from retinal and cortical projections [1,22]. These terminals were visualized by immunostaining with antibodies against Vesicular Glutamate Transport 1 (VGluT1) and VGluT2, which label cortical and retinal nerve terminals, respectively [12,36]. Striking differences were observed in both VGluT1- and VGluT2-IR in visual thalamic nuclei. VGluT1-IR was dense in dLGN making it difficult to delineate individual synaptic terminals (Figure 2A,C). This result was not entirely surprising given previous estimates that 30 to 50% of all terminals in the dorsal thalamic nuclei originate from cortex [3,42].

In contrast to the dense cortical inputs in dLGN, VGluT1-positive terminals sparsely populated vLGN (Figure 2A,D). To test whether VGluT1-containing cortical terminals in dLGN and vLGN originated from distinct cortical layers, we examined cortical projections in *Golli-tau-gfp* transgenic mice, in which layer VI cortical neurons (but not layer V neurons) are labeled with Green Fluorescent Protein (GFP) [35,36,42]. As was the case for VGluT1-IR, tau-GFP distribution was so dense in adult dLGN that individual nerve terminals could not be distinguished, even at high magnification in single optical sections of confocal images (Figure 2E,G). In fact, the only regions of dLGN in *Golli-tau-gfp* transgenic mice that appeared devoid of GFP (in single optical sections) were regions containing cell somas, VGluT2-IR retinal terminals [12], or blood vessels (Figure 2G,H and JS and MAF, unpublished observations). In contrast, tau-GFP-positive projections were absent from vLGN (Figure 2F). This suggested that VGluT1-positive terminals in mouse vLGN do not arise from cortical layer VI. Alternative possibilities were that VGluT1-positive terminals in vLGN arose from cortical layer V [24,43], from retinal projections, or from the superior colliculus, a third source of glutamatergic inputs to visual thalamus [24]. We ruled out the possibility that VGluT1-positive terminals arose from retinal projections since they persisted in vLGN of *Math5*^*-/-*^ mutant mice, which lack retinal inputs to the thalamus (JS and MAF, unpublished observations) [36,44,45]. It was also unlikely that VGluT1-positive terminals in vLGN arose from superior colliculus since these neurons express VGluT2 and not VGluT1 [11]. Taken together with studies in rat thalamus, these data lead us to speculate that VGluT1-positive terminals in mouse vLGN originate from layer V cortical neurons [24,43]. In contrast to modulatory inputs that originate from cortical layer VI, inputs from layer V generate strong, driver-like input to higher order thalamic nuclei [1,46,47]. This raises the possibility that VGluT1-positive corticogeniculate synapses provides a primary excitatory drive for vLGN neurons.

Like VGluT1-IR, the distribution of VGluT2-IR differed significantly in distinct nuclei of the mouse visual thalamus. Little immunoreactivity was observed in IGL or in the non-retino-recipient regions of vLGN (i.e. the internal division of the vLGN) [48]. Robust VGluT2-IR was detected in both vLGN and dLGN, however the patterns of immunoreactivity in these nuclei differed. As noted previously, the size of VGluT2-containing terminals varied between these regions, with larger terminals predominating in dLGN (Figure 2I-L) [48]. Not only were dLGN VGluT2-positive terminals larger than their vLGN counterparts, but they appeared larger than all other types of terminals analyzed here (for example, in dLGN VGluT2-positive terminals averaged 2.43 μm^2^ ± 0.38 μm^2^ (SD) whereas VAChT-positive terminals averaged 0.70 μm^2^ ± 0.06 μm^2^ and GAD67-positive terminals averaged 1.21 μm^2^ ± 0.11 μm^2^; see Figure 2K,L). Interestingly, despite terminals being dramatically larger in dLGN, a significantly larger fraction of each confocal image was occupied by VGluT2-IR in vLGN (15.6% ± 0.5% of vLGN images were occupied by VGluT2-IR versus 8.3% ± 0.3% in dLGN; *P* <0.0001 by Student’s *t*-test).

Based on previous reports showing VGluT2 expression by RGCs [12], we interpreted these findings to indicate that retinal terminals are smaller but more densely distributed in vLGN. To ensure that VGluT2-containing terminals originated from the retina, we assessed VGluT2-IR in *Math5*^*-/-*^ mutant mice. Few VGluT2-positive terminals were observed in *Math5*^*-/-*^ mutant vLGN and dLGN. We interpret this data to indicate that: 1). The vast majority of VGluT2-positive terminals in mouse visual thalamus were retinal terminals; 2). A small cohort of nonretinal, VGluT2-positive terminals existed in both vLGN and dLGN. These terminals, which we suspect arise from superior colliculus (based on the robust expression of VGluT2 by collicular projection neurons) [11], appeared similar in size in both vLGN and dLGN and are smaller than dLGN retinal terminals.

### Nuclei-specific differences in retinal terminal morphology in mouse visual thalamus

We interpret differences in VGluT2-IR in vLGN and dLGN to reflect differences in retinal nerve terminal morphology in these regions. However, since VGluT2-IR labeled synaptic vesicle pools and not entire nerve terminals, an alternative possibility was that the quantity or distribution of synaptic vesicles (and not terminal size) differed in vLGN and dLGN. To distinguish between these possibilities we labeled retinal axons and their terminals with two orthologous techniques: transgenic expression of fluorescent reporter proteins and anterograde labeling by intraocular injection of fluorescently conjugated tracer molecules.

**Figure 3:**
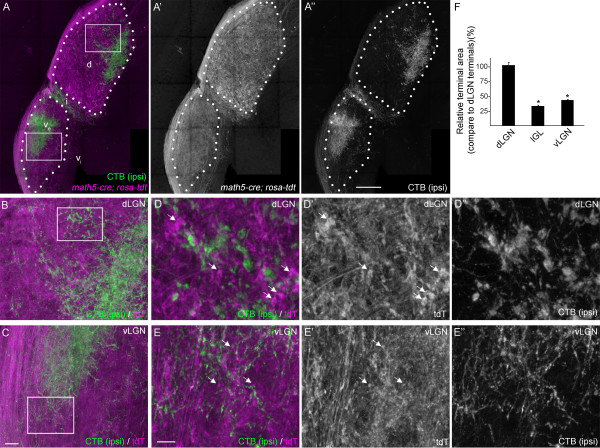
**Genetic labeling of retinal terminals in subnuclei of mouse visual thalamus. A**. Retinal projections (magenta) were labeled by crossing *Rosa- tdt* reporter mice with *Math5-cre* driver mice. Few (if any) cells in the thalamus express tdTomato (tdT) in *Math5-cre; Rosa-tdt* transgenic reporter mice. Ipsilateral retinal projections (green) were co-labeled anterogradely by intraocular injection of AlexaFluor488-conjugated cholera toxin subunit B (CTB) in the ipsilateral eye. Outlines of dorsal lateral geniculate nucleus (dLGN) and the external division of ventral lateral geniculate nucleus (vLGN) are depicted with white dots. d, dLGN; v_e_, external division of vLGN; v_i_, internal division of vLGN; i, IGL. White boxes depict regions enlarged in **B** and **C**. **B,C**. High magnification images of tdTomato (tdT; magenta) labeled retinal projections and CTB-labeled ipsilateral retinal projections. **D, E**. High magnification images of regions of dLGN **(D)** and vLGN **(E)** highlighted by the white boxes in **B** and **C,** respectively. Arrows highlight tdT-containing retinal terminals in dLGN and vLGN. All images are maximum projection confocal images. **F**. Relative tdT-labeled retinal terminal size (compared to retinal terminals in dLGN) was quantified in single optical sections of dLGN, vLGN and IGL. Terminal sizes in IGL and vLGN were statistical smaller than those in dLGN (*P* <0.001 by Neuman-Keuls Test), but were not statistically different from each other (*P* = 0.23). Scale bar in **A** = 200 μm, in **C** = 25 μm for **B,C**, in **E** = 10 μm for **D,E**.

To fluorescently label retinal axons transgenically, we employed a Cre-Lox recombination-based approach by crossing *Math5-cre* mice (in which Cre Recombinase (Cre) expression is largely restricted to retinal neurons) with *Rosa-stop-tdt* reporter mice (in which the fluorescent reporter protein tdTomato (tdT) is generated only in cells containing Cre). In *Math5-cre*; *Rosa-stop-tdt* mice, tdT was robustly distributed within the optic nerve, chiasm and tract and in axonal arbors in all retino-recipient nuclei (Figure 3 and GLC, AM, and MAF, unpublished observations). We examined tdT-containing terminal arbors at high magnification in vLGN, IGL, and dLGN (Figure 3B-E). TdT-positive terminal areas were measured in single optical sections from high magnification confocal images. Similar to results with VGluT2-IR, tdT-containing terminals were significantly larger in dLGN than in adjacent regions of mouse visual thalamus (Figure 3F). TdT-containing terminals were quantitatively similar in size in vLGN and IGL (Figure 3A,F).

**Figure 4:**
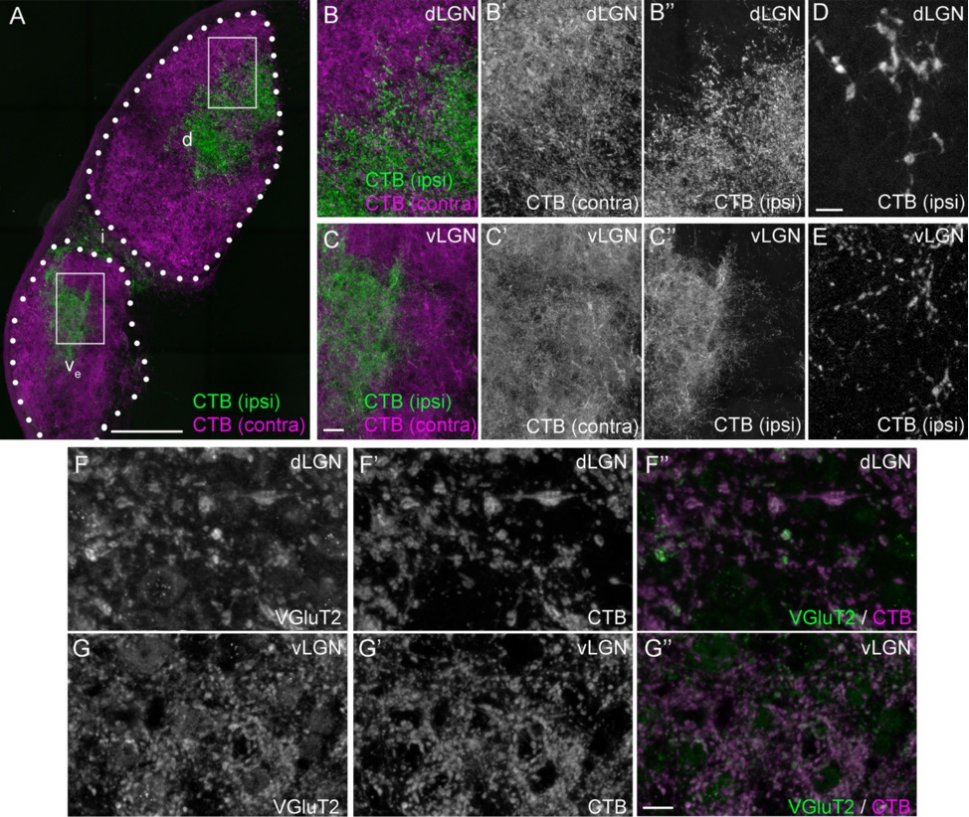
**Anterograde labeling of retinal terminals in subnuclei of mouse visual thalamus. A**. Retinal projections in P35 wild-type mice were labeled by intraocular injection of fluorescently conjugated cholera toxin subunit B (CTB). Left eyes were injected with Alexa Fluor 555 CTB (magenta) and right eyes were injected with Alexa Fluor 488 CTB (green). LGN from right hemispheres are shown. ‘Contra’ denotes projections originating from the contralateral retina and ‘ipsi’ denotes projections originating from the ipsilateral retina. Outlines of dorsal lateral geniculate nucleus (dLGN) and the external division of ventral lateral geniculate nucleus (vLGN) are depicted with white dots. d, dLGN; v_e_, external division of vLGN; i, IGL. White boxes depict regions enlarged in **B** and **C**. **B,C**. High magnification images of CTB-labeled retinal terminals in dLGN **(B)** and vLGN **(C)**. Note the punctate appearance of retinal projections in dLGN and the denser and diffuse labeling of retinal terminals in vLGN. **D,E**. High magnification image of ipsilateral retinal projections from **B,C** depict differences in retinal terminal size in dLGN **(D)** and vLGN **(E)**. **F,G**. Immunolabeling CTB-labeled tissue with antibodies against VGluT2 demonstrated that CTB was enriched at synaptic sites in LGN and confirmed that VGluT2-positive terminals were derived from retinal projections. All images are maximum projection confocal images. Scale bar in **A** = 200 μm, in **C** = 25 μm for **B,C**, in **E** = 10 μm for **D,E**, and in **G** =10 μm for **F,G**.

**Figure 5:**
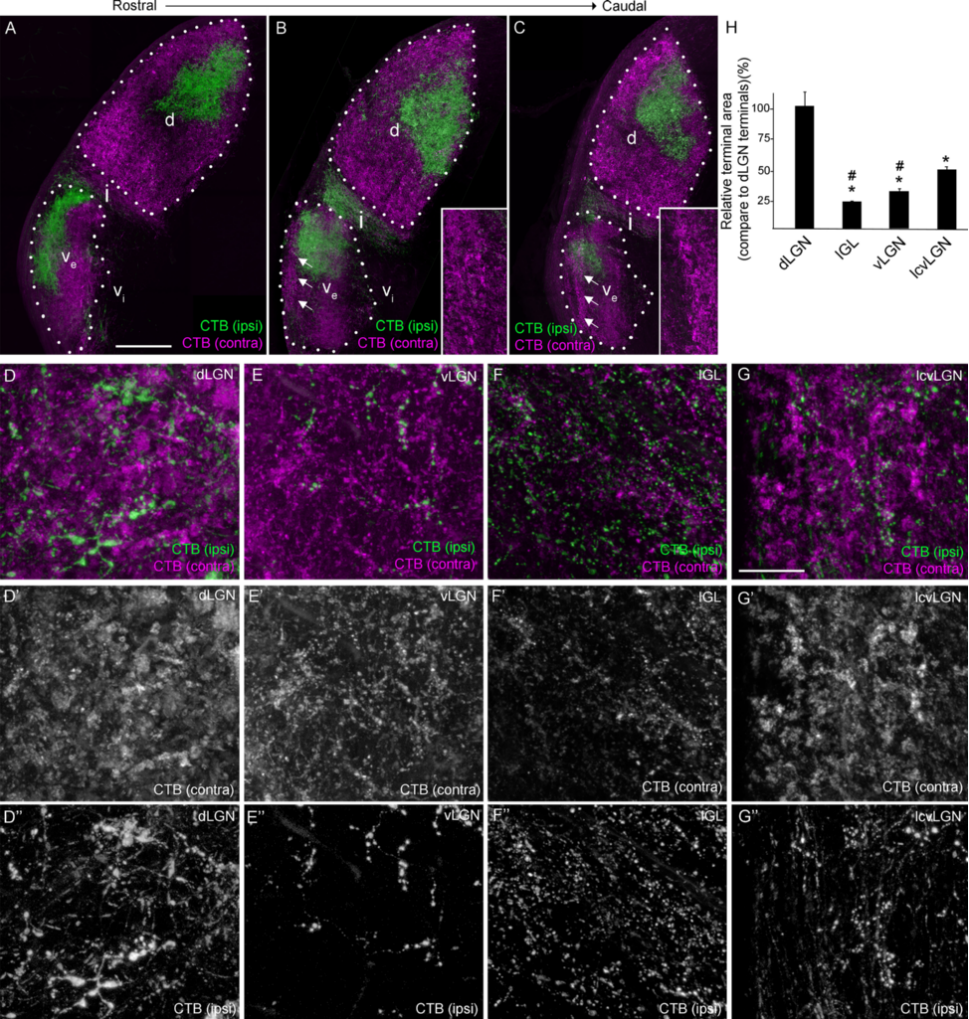
**Laminar-specific differences in retinal terminal morphology in ventral lateral geniculate nucleus (vLGN). A-C**. Retinal projections in P35 wild-type mice were labeled by intraocular injection of fluorescently conjugated cholera toxin subunit B (CTB). Left eyes were injected with Alexa Fluor 555 CTB (magenta) and right eyes were injected with Alexa Fluor 488 CTB (green). ‘Contra’ denotes projections originating from the contralateral retina and ‘ipsi’ denotes projections originating from the ipsilateral retina. Three sections of lateral geniculate nucleus (LGN) from different rostral to caudal regions of the right hemispheres are shown. In more caudal sections, a small, lateral region of vLGN emerges that contains retinal terminals that appear to share characteristics of dorsal lateral geniculate nucleus (dLGN) retinal terminal morphology (arrows). Insets in **B,C** show high magnification images of this regions, which we term the lateral shell of caudal vLGN (lcvLGN). ‘Contra’ denotes projections originating from the contralateral retina and ‘ipsi’ denotes projections originating from the ipsilateral retina. Outlines of dLGN and the external division of vLGN are depicted with white dots. d, dLGN; v_e_, external division of vLGN; v_i_, internal division of vLGN; i, IGL. **D-G**. High magnification images of CTB-labeled retinal terminals in dLGN **(D)**, external division of the vLGN **(E)**, IGL **(F)**, and lcvLGN **(G)**. All images are maximum projection confocal images. **H**. Relative CTB-labeled retinal terminal areas (compared to retinal terminals in dLGN) were quantified in single optical sections of dLGN, vLGN, IGL and lcvLGN. Retinal terminal sizes in IGL, vLGN and lcvLGN were statistical smaller than those in dLGN (*P* <0.0001 by Neuman-Keuls Test). CTB-labeled terminal sizes in IGL and vLGN were also significantly smaller than those in lcvLGN (*P* <0.02 by Neuman-Keuls Test), but were not statistically different from each other (*P* = 0.48). Scale bar in **A** = 200 μm for **A-C** and in **G** = 25 μm for **D-G**.

As an alternative approach, we next examined retinal terminals by anterogradely labeling all retinal projections with intraocular injections of fluorescently conjugated cholera toxin subunit B (CTB) (Figures 4 and 5) [49]. Retinal projections from each eye were differentially labeled with distinct fluorescently conjugated versions of CTB. This approach confirmed results reported above: striking differences were observed in the size and distribution of CTB-labeled terminals in vLGN and dLGN. First, CTB-labeled terminals were significantly larger in dLGN than in vLGN and IGL (Figures 4A-E, 5H). Qualitatively, the distribution of CTB-labeled terminals appeared denser in vLGN than in dLGN (see the diffuse appearance of CTB-labeling in vLGN in Figure 4C versus the coarse appearance in dLGN in Figure 4B); however, since CTB labeled both retinal axons and terminals, it remained possible that smaller-CTB labeled elements represented axons in cross section rather than terminals. For this reason, we immunostained CTB-labeled tissue for VGluT2. In both vLGN and dLGN, CTB faithfully co-localized with VGluT2, indicating that the overwhelming majority of these structures in the adult mouse vLGN and dLGN were retinal terminals and not axonal shafts (Figure 4F,G). As an aside, this was somewhat surprising since CTB is regularly used to label axonal projections in many brain regions. However, comparison of CTB-labeled material with tdT-labeled material from *Math5-cre*; *Rosa-stop-tdt* mice showed clear differences in axonal shaft labeling and indicate that CTB labeled retinal terminals more robustly than axon shafts (Figures 3, 4 and 5: compare optic tract labeling versus terminal arbor labeling in both methods).

Retino-recipient nuclei in a wide variety of mammalian brains are subdivided into discrete laminae (or regions), a feature that facilitates the flow of visual information through distinct parallel pathways in the brain. Although laminae are not readily identifiable by cyto-architectural analysis, it is becoming increasingly clear that retinal terminals are targeted into ‘hidden’ laminae of the rodent dLGN ([50-52]) In mice, these ‘hidden’ subdivisions occur along the lateral-medial axis and are referred to as the ‘shell’ and ‘core’ of dLGN [52]. As we analyzed retinal terminal size throughout LGN, we noted a small, lateral ‘shell’-like region of vLGN that contained retinal terminals that were morphologically distinct from the rest of vLGN and IGL (Figure 5A-G). This morphologically distinct region of vLGN was present only in caudal sections of vLGN; therefore, we refer to it as the lateral shell of caudal vLGN (lcvLGN). CTB-labeled terminals in lcvLGN were significantly larger than those in IGL, in more medial ‘core’-like regions of caudal vLGN, and in both medial and lateral regions of rostral vLGN (Figure 5H). An interesting feature of terminals in lcvLGN that we failed to detect in any other region of the visual thalamus (or other retino-recipient nuclei) was that retinal terminal size correlated with eye of origin. Terminals in the lcvLGN that originated from the contralateral retina appeared consistently larger than those in other regions of vLGN of IGL (Figure 5E-G). In contrast, terminals in the lcvLGN that originated from the ipsilateral retina appeared small and consistent in size to the more typical retinal terminals in IGL and vLGN terminals (Figure 5E-G). At least two classes of direction-selective RGCs have been identified that not only project to both dLGN and lateral regions of caudal vLGN, but exclusively project to contralateral thalamus [27,53]. It is possible that axons from these classes of direction-selective RGCs generate these large terminals in lcvLGN.

**Figure 6:**
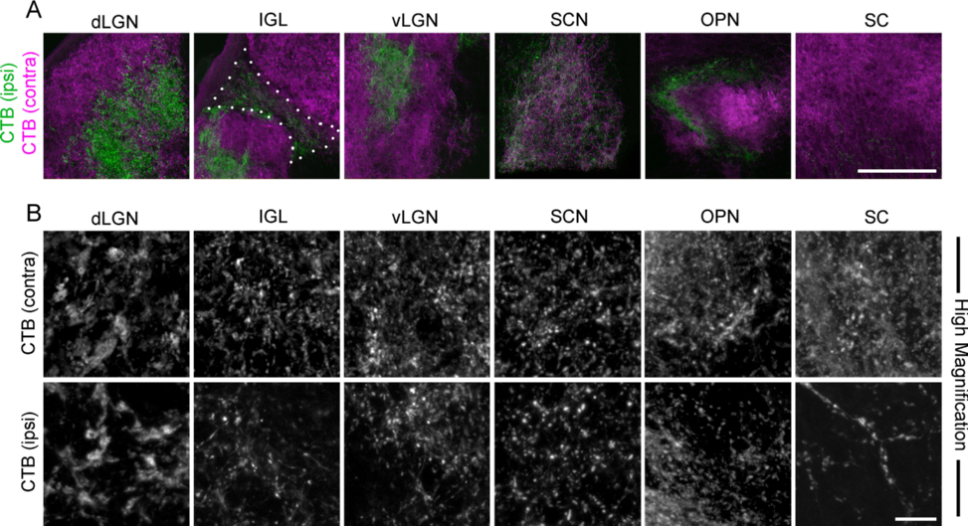
**Anterograde labeling of retinal terminals in other retino-recipient nuclei. A**. Retinal projections in P35 wild-type mice were labeled by intraocular injection of fluorescently conjugated cholera toxin subunit B (CTB). Left eyes were injected with Alexa Fluor 555 CTB (magenta) and right eyes were injected with Alexa Fluor 488 CTB (green). Lateral geniculate nucleus (LGN) from the right hemispheres is shown. ‘Contra’ denotes projections originating from the contralateral retina and ‘ipsi’ denotes projections originating from the ipsilateral retina. Confocal images were acquired from the dorsal lateral geniculate nucleus (dLGN), intergeniculate nucleus (IGL), ventral lateral geniculate nucleus (vLGN), suprachiasmatic nucleus (SCN), olivary pretectal nucleus (OPN), and superior colliculus (SC). IGL is outlined by white dots. **B**. High magnification images of both contralateral and ipsilateral retinal projections to each region are shown (note - contralateral and ipsilateral panels are not all from the same image). Scale bar in A = 200 μm and in B = 20 μm.

Finally, having thoroughly examined terminal morphology in visual thalamic nuclei by confocal microscopy, we next examined how retinal terminal morphology differed in non-thalamic retino-recipient nuclei. We assessed CTB-labeled terminals in the superior colliculus (SC), a midbrain structure targeted by most retinal axons target in mice, and two accessory visual system nuclei, the suprachiasmatic nucleus (SCN) and the olivary pretectal nucleus (OPN). CTB-labeled retinal terminals in SC, OPN, and SCN appeared morphologically similar to vLGN and IGL terminals, and appeared smaller than those in dLGN (Figure 6). These results are not entirely surprising since previous measures of retinal terminal size in mouse SC are comparable to our measures of CTB- or VGluT2-labeled terminals in vLGN and IGL [54].

### Ultrastructural analysis of retinal terminals in mouse visual thalamus

Retinal terminals are clustered into dense ‘synaptic islands’ (i.e. glomeruli) in dLGN. Whether such complex arrangements exist in mouse vLGN remains unresolved (but see [55]). Therefore, one possibility is that dLGN retinal terminals appeared larger following immunohistochemistry (IHC), genetic labeling or anterograde labeling due to the inability of confocal microscopy to fully resolve individual terminals in these complex synaptic arrangements. To address this possibility, we examined retinal terminal morphology with serial block face scanning electron microscopy (SBFSEM), a technique that permits the tracing of ultrastructural morphology of nerve terminals through relatively large volumes of serially sectioned tissue. Four samples of vLGN and dLGN from adult wild-type mice were analyzed by SBFSEM. Each sample set represented a 40 μm by 40 μm by 15 μm volume of tissue.

**Figure 7:**
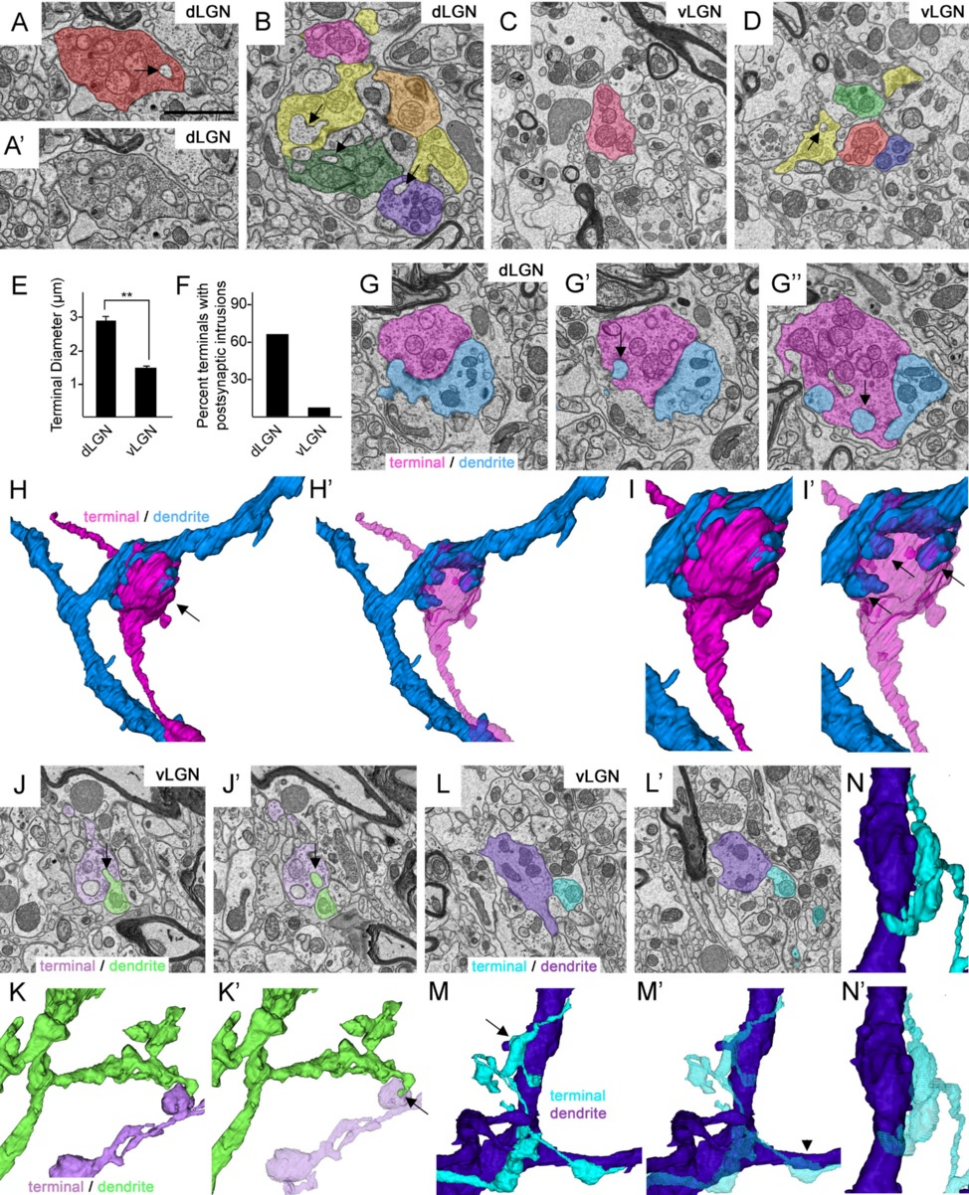
**Ultrastructural analysis of retinal terminals in subnuclei of mouse visual thalamus.** Retinal terminals were identified in serial block face scanning electron microscopy (SBFSEM) micrographs based upon their pale mitochondria and round, abundant synaptic vesicles. **A,B**. Retinal terminals in P42 dorsal lateral geniculate nucleus (dLGN). **A’** shows non-pseudo-colored image depicted in **A**. **C,D**. Retinal terminals in P42 ventral lateral geniculate nucleus (vLGN). Each terminal and axon traced was given a unique color; therefore terminals labeled the same color belong to the same axonal arbor. Retinal terminals in dLGN contain a large number of processes from dendrites that extend into the nerve terminals (compare arrows in **A,B** with those in **D)**. **E**. Retinal terminals in dLGN are statistically larger than those in vLGN (*P* <0.000001 by Student’s *t*-test; n = 3 datasets containing a total of 111 terminals in dLGN and 148 in vLGN). **F**. Quantification of the percent of terminals traced that contain postsynaptic intrusions (see arrows in **A,B,D**,**G,** and **J**). **G-I**. SBFSEM micrographs demonstrate the complex nature of terminal-dendrite interactions in dLGN. A single retinal terminal is labeled in pink and its dendritic partner is labeled in blue. In **G’** and **G”**, arrows highlight dendritic projections into the retinal terminal. **H** and **H’** depict 3D reconstructions of the retinal terminal and dendrite labeled in **G**. **I,I’** represents a high magnification image of the terminal-dendrite interface in **H**. The retinal terminal has been made translucent in **H’** and **I’**. 3 finger-like dendritic protrusions that invade a single terminal bouton are highlighted with arrows in **I’. J-N**. Multiple SBFSEM micrographs through two retinogeniculate synapses demonstrate the less complex nature of terminal-dendrite interactions in vLGN. **K** and **M** depict 3D reconstructions of the retinal terminals and dendrites traced in **J** and **L** respectively. Arrows in **J** and **K’** indicate a small dendritic protrusion that extends into the retinal terminal. In **M,** 4 terminals from the same axon contact the purple dendrite and none contain dendritic protrusions. **N** shows a high magnification, rotated image of the terminal and dendrite in indicated by the arrow in **M**. Note the absence of dendritic protrusions into this terminal bouton. Retinal terminals and axons been made translucent in **K’,M’** and **N’**. Scale bar in **A** = 1.5 μm for **A-D**, **G**, **J**, and **L**.

**Figure 8:**
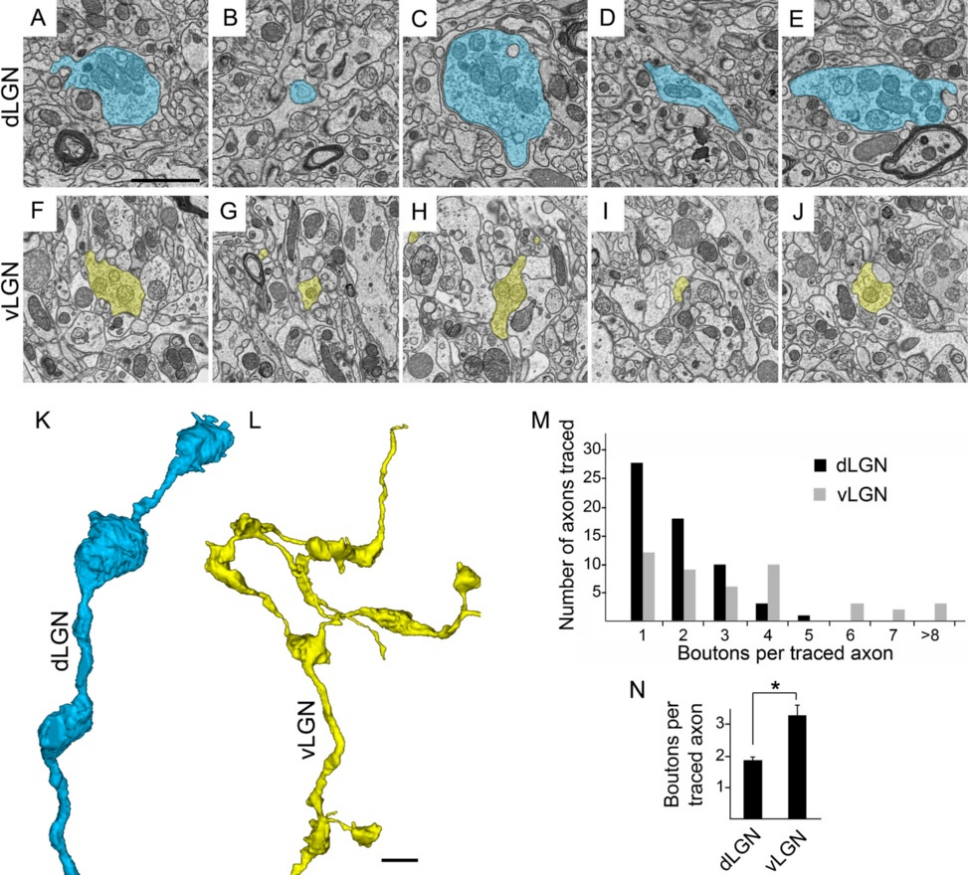
**Ultrastructural analysis of numbers of terminals per retinal axon in subnuclei of mouse visual thalamus. A-J**. Serial block face scanning electron microscope (SBFSEM) micrographs show multiple terminal boutons and axons from a single retinal axon in dLGN **(A-E)** and vLGN **(F-J)**. K, L. 3D reconstructions of axonal arbors from **A-E** (see **K**) and **F-J** (see **L**). These reconstructions are at the same scale and represent entire axonal arbors from dorsal lateral geniculate nucleus (dLGN) and ventral lateral geniculate nucleus (vLGN) datasets that were 40 μm by 40 μm by 15 μm. **M**. Numbers of terminal boutons were counted in each axon traced in dLGN and vLGN (n = 3 datasets per tissue regions; 59 axons in dLGN, 44 axons in vLGN). Retinal axons in dLGN datasets contain fewer terminal boutons than those in vLGN. **N**. Quantitation of the mean numbers of terminal boutons per axon in dLGN and vLGN. Axons traced in dLGN contain a significantly smaller number of terminal boutons than those in vLGN (*P* <0.00005 by Student *t*-test). Scale bar in **A** = 1 μm for **A-J** and in **L =** 1.5 μm for **K,L**.

Since retinal terminals were unlabeled in SBFSEM datasets, we used ultrastructural morphology - specifically, the presence of round synaptic vesicles and pale mitochondria - to identify retinal terminals. In dLGN, pale-mitochondria containing terminals (i.e. RLPs) were significantly larger than adjacent terminals containing darkly stained mitochondria (Figures 7A,B,G and 8A-E)(see also [6,14]). As previously reported [6,14], dLGN RLPs made multiple synaptic contacts, contained well-defined nonsynaptic adherent junctions, clustered into terminal-rich synaptic islands, and were often encapsulated into glomeruli by glial cells (Figures 7A,B,G and 8A-L). Surprisingly, tracing arbors associated with each terminal in RLP-rich synaptic islands revealed that many of these terminals originated from distinct retinal axons (Figure 7B). In a few cases, we observed RLPs from 4 to 6 different axons synapsing onto the same dendrite (see Figure 7B). However, based on single retinogeniculate axon tracing studies by Crair and colleagues [56], it remains possible that these RLPs originated from distant branches of just one or two retinal axons, but that that these branches occurred outside of the volume of tissue in SBFSEM datasets.Another hallmark feature of dLGN RLPs observed in our analyses were postsynaptic, finger-like protrusions, from both relay neuron dendrites and F terminals, that extended into retinal terminals (see arrows in Figure 7A,B,G). While such structures had been previously identified at single, encapsulated RLPs, we observed them frequently in both single and clustered RLPs. In fact >60% of dLGN RLPs analyzed contained postsynaptic finger-like protrusions (Figure 7F). In many cases a single dendrite extended multiple finger-like protrusions into a single RLP, a feature that was only fully appreciated following the 3D reconstruction of terminals and dendrites in their entirety in the SBFSEM dataset (Figure 7G-I).

We used identical criteria to characterize the ultrastructural morphology of retinal terminals in vLGN. Similar to dLGN, pale mitochondria-containing terminals made multiple synaptic contacts and were closely associated with inhibitory terminals (Figure 7J and SH, JSB, and MAF, unpublished observation). Pale mitochondria-containing retinal terminals in vLGN also existed in isolation or in clusters around dendrites, although these clusters were often not encapsulated by glial cells as was the case in dLGN (Figure 7C,D). However, despite these similarities, retinal terminals appeared strikingly different in vLGN. First, they appeared significantly smaller in vLGN than dLGN (Figures 7 and 8). After tracing each pale-mitochondria containing terminal in its entirety, we measured the diameter of the widest portion of each presynaptic bouton. The mean width of retinal terminals in dLGN was 2.88 μm ± 0.11 μm versus 1.55 μm ± 0.04 μm in vLGN (*P* <0.00001 by Student’s *t*-test. n = 148 boutons in vLGN; n = 111 boutons in dLGN. Data are mean ± SEM) (Figure 7E). Second, retinal terminals in vLGN appeared less morphological complex, as <10% of these terminals contained finger-like invaginations from postsynaptic target cells (Figure 7F,J-N). These results are in line with previous studies in cats, which revealed smaller retinal terminals and a lack of typical, glial encapsulated glomeruli in vLGN [55].

In serially tracing entire retinal arbors with SBFSEM datasets, we observed one addition difference in presynaptic axons and boutons in these nuclei. Axonal arbors traced in vLGN (n = 44) contained on average more terminal boutons than axons traced in dLGN (Figure 7). Figure 7M shows a distribution plot of the numbers of terminal boutons in each axon traced in vLGN and dLGN. Axons in dLGN contained approximately 40% fewer terminal boutons than those traced in vLGN (1.85 ± 0.12 boutons per axon in dLGN (n = 59 axons) versus 3.29 boutons per axon in vLGN (n = 44). Data are mean ± SEM. *P* <0.00005 by Student’s *t*-test).

### Distinct patterns of retinogeniculate transmission in ventral lateral geniculate nucleus and dorsal lateral geniculate nucleus

At glutamatergic synapses, a structure-function relationship has been described in which nerve terminal size significantly influences the functional strength of information transfer [47]. Anatomically large glutamatergic synapses with little convergence on postsynaptic target cells (for example, dLGN RLPs) produce large amplitude excitatory postsynaptic currents (EPSCs), while smaller terminals elicit weaker postsynaptic responses. Based on results described above, we postulated that the synaptic strength of retinogeniculate synapses should vary between mouse vLGN and dLGN.

**Figure 9:**
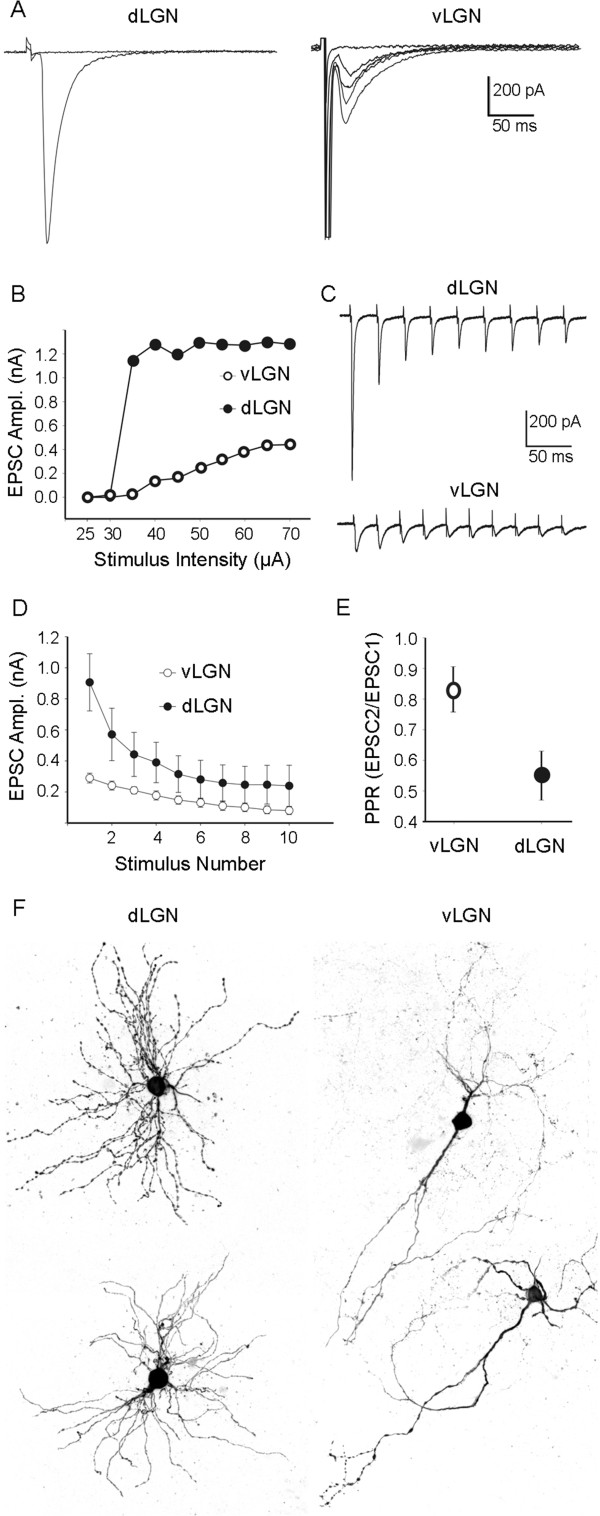
**Glutamatergic synaptic responses evoked by optic tract stimulation in subnuclei of mouse visual thalamus. A**. Examples of synaptic responses in P35 dorsal lateral geniculate nucleus (dLGN) and ventral lateral geniculate nucleus (vLGN) neurons. Synaptic responses in dLGN neurons show all-or-none and larger amplitude excitatory postsynaptic currents (EPSC), whereas vLGN neurons show graded responses with smaller amplitude EPSCs. **B**. Population data reveal that EPSC amplitudes were smaller in vLGN and progressively increasing stimulus intensity increased peak amplitudes of EPSCs in vLGN but not dLGN. This suggests that while dLGN relay neurons are innervated by only one or two retinal axons, vLGN cells receive smaller inputs from larger numbers of retinal axons. **C**. Differences in pair-pulse depression (PPD) were observed at retinogeniculate synapses in dLGN and vLGN. An example of synaptic responses recorded in dLGN and vLGN neurons following a train of stimuli with a 25 ms interstimulus interval is shown in **C**. Examples of current traces showing differences in pair-pulse depression at retinogeniculate synapses in dLGN and vLGN. Synaptic responses recorded in dLGN and vLGN neurons following a train of stimuli with a 50 ms interstimulus interval. **D**. Average peak EPSC amplitudes in dLGN and vLGN following trains of stimuli (20 Hz, 10 pulses). **E**. The paired pulse ratio was plotted using the peak EPSC amplitudes following the first and second stimuli. Thus, in addition to exhibiting weaker postsynaptic responses, retinogeniculate synapses in vLGN show weaker paired pulse depression compared to those in dLGN. **F**. Examples of reconstructions of biocytin-filled relay neurons in dLGN and vLGN.

To address this, we performed *in vitro* whole cell patch recordings from acutely prepared thalamic slices to measure excitatory synaptic responses evoked by optic tract stimulation in P35 vLGN and dLGN neurons. Representative excitatory postsynaptic currents (EPSCs) from each region are shown in Figure 9A. In dLGN, optic tract stimulation evoked large amplitude EPSCs that showed little variation in size to a progressive increase in stimulus intensity (Figure 9A,B). Such all-or-none type events reflect little or no retinal axon convergence onto dLGN relay neurons [49,57]. As is the case for other large, excitatory terminals [47], retinogeniculate synaptic responses evoked by repetitive stimulation showed strong depression in dLGN. For example, EPSCs decreased in amplitude with each successive stimulus pulse of a train delivered at 20 Hz (Figure 9C-E). To further quantify the degree of synaptic depression, we generated paired pulse ratios (EPSC2/EPSC1) in which the amplitude of the initial response was compared to the second one. As expected, dLGN cells showed strong paired pulse depression with amplitude of initial EPSC twofold larger than the second (Figure 9E). These retinally evoked synaptic profiles have been well documented and are the hallmark features of driver-like or Class 1 glutamatergic synapses [1,47].

By contrast, responses in vLGN evoked by optic tract stimulation were fivefold smaller (Figure 9A). Moreover, EPSCs showed a graded increase in amplitude with a progressive increase in stimulus intensity (Figure 9B), indicating a higher degree of retinal convergence on vLGN neurons [47]. This result may, at least in part, explain the increased density of retinal terminals observed following VGluT2-IHC and CTB labeling in vLGN (Figures 2, 4 and 5) and the increased number of boutons per retinal axon observed with SBFSEM in vLGN (Figure 8). In contrast to dLGN, retinogeniculate synapses in vLGN exhibit much less depression following repetitive optic tract stimulation (Figure 9C-E).

Taken together, anatomical and physiological comparisons between dLGN and vLGN suggest that retinal terminals in vLGN do not seem to possess the hallmark features of a driver-like synapse. Instead, they seem to have features consistent with modulatory or Class 2-type synapses. Retinal terminals in vLGN appear smaller in size than those in dLGN, produce weak EPSCs, and exhibit higher levels of convergence on vLGN neurons. Moreover, studies in rodents further suggest that retinal terminals contact distal regions of vLGN neuron dendrites [58] whereas those in dLGN reside just proximal to somata [20]. While all of these features more closely resemble features associated with modulatory glutamatergic inputs rather than driver synapses, it is important to note that vLGN synapses responses did not show paired pulse facilitation, a feature common to most modulatory synapses (Figure 9E). Thus, perhaps retinal synapses in vLGN represent a hybrid between these two terminal types.

### Development of retinal terminal morphology in ventral lateral geniculate nucleus and dorsal lateral geniculate nucleus

**Figure 10:**
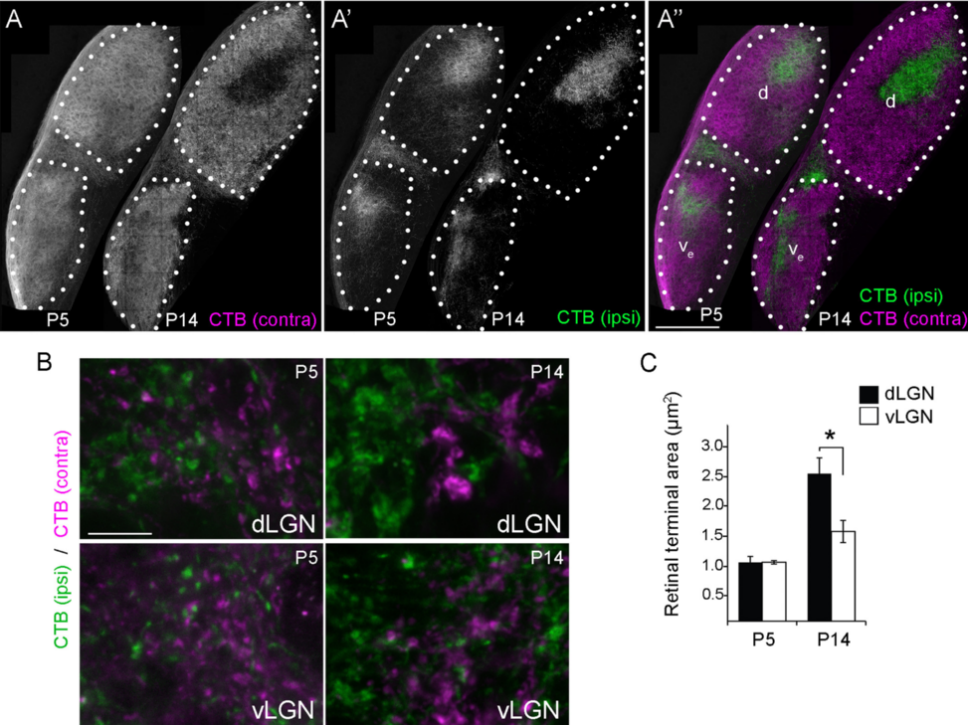
**Anterograde labeling of retinal terminals in developing subnuclei of mouse visual thalamus. A**. Retinal projections in P5 and P14 wild-type mice were labeled by intraocular injection of fluorescently conjugated cholera toxin subunit B (CTB). Left eyes were injected with Alexa Fluor 555 CTB (magenta) and right eyes were injected with Alexa Fluor 488 CTB (green). LGN from right hemispheres are shown. ‘Contra’ denotes projections originating from the contralateral retina and ‘ipsi’ denotes projections originating from the ipsilateral retina. Outlines of dorsal lateral geniculate nucleus (dLGN) and the external division of ventral lateral geniculate nucleus (vLGN) are depicted with white dots. d, dLGN; v_e_, external division of vLGN; i, IGL. **B**. High magnification images of CTB-labeled retinal terminals in dLGN and vLGN at P5 and P14. **C**. CTB-labeled retinal terminal areas were quantified in single optical sections of dLGN and vLGN at P5 and P14. Retinal terminal sizes in vLGN and dLGN were not statistically different from each other at P5 but statistically different at P14 (*P* <0.0001 by the Tukey Kramer test for differences between means). All images are maximum projection confocal images. Scale bar in **A** = 200 μm and in **B** = 15 μm.

We recently documented the morphological enlargement of retinal terminals in mouse dLGN during the first 2 weeks of postnatal development [6,59]. Therefore, we next asked whether differences in terminal morphology were apparent from the onset as retinogeniculate synapses formed, or whether they undergo unique developmental changes that transform them. To answer this question we anterogradely labeled retinal projections with CTB at P5 and P14. At P5, retinal axons have differentiated into immature terminals and are beginning to undergo activity-dependent refinement, whereas at P14, retinogeniculate circuit refinement is largely complete and terminals are functionally maturing into their adult phenotype [49,60,61]. CTB-labeling revealed that the size of retinal terminals in P5 vLGN and dLGN was indistinguishable (Figure 10) (at P5, dLGN CTB-positive terminals averaged 1.10 μm^2^ ± 0.20 μm^2^ whereas vLGN terminals averaged 1.10 μm^2^ ± 0.09 μm^2^. Data are shown as SEM; see Figure 10). By P14, statistical differences in terminal sizes in these nuclei emerged (at P14, dLGN CTB-positive terminals averaged 2.75 μm^2^ ± 0.34 μm^2^ whereas vLGN terminals averaged 1.67 μm^2^ ± 0.24 μm^2^. Data are shown as SEM. *P* <0.0002 by the Tukey Kramer test for differences between means; see Figure 10). Thus, retinal terminals in vLGN and dLGN are initially similar in size but undergo nuclei-specific transformations as they develop their adult-like morphologies.

At present it remains unclear whether differences in terminal development in vLGN and dLGN are intrinsic characteristics of the classes of neurons that project to either nuclei or whether target-derived cues differentially transform terminals in nuclei-specific fashions. A case could be made that terminal morphology is an intrinsic feature of axons if the larger retinal terminals observed in lcvLGN do in fact originate from the same direction selective RGCs that project to lateral regions of dLGN [27,53]. However, both single axon tracing studies and observations of genetically labeled RGCs indicate that dLGN-projecting retinal axons branch to innervate other retino-recipient nuclei that lack anatomically large retinal terminals [33,53,56]. This suggests that identity alone does not predetermine terminal size.

An elegant series of experiments by Frost and colleagues supports the notion of target-derived cues capable of differentially shaping retinal terminal maturation and morphology in different thalamic nuclei [62-65]. In these studies, the misrouting of retinal axons into non-retino-recipient regions of dorsal thalamus resulted in retinal terminals adopting nonretinal-like morphologies [64,65]. In fact, data from our own studies in mice lacking canonical Reelin receptors [48] support the notion that local environmental cues released by nuclei-specific cells pattern the targeting and differentiation of nerve terminals in mouse visual thalamus. At present, the nature of such cues remains unclear.

## Conclusions

In the present study, we examined the composition, distribution and morphology of nerve terminals in three adjacent, retino-recipient nuclei in the mouse thalamus. Results demonstrate that despite all receiving, processing and relaying light-derived signals from the retina, each nucleus contains distinct sets of neurochemically-defined nerve terminals. Inhibitory terminals (which contained GAD67, GAD65, or VGAT) are most abundant in vLGN. Cortical inputs are most abundant in dLGN and appear to originate from a different layer of cerebral cortex in each visual thalamic nucleus. And most surprising was our discovery that retinal terminals vary significantly in their abundance and morphology in these nuclei. While retinal terminals appear significantly larger and more complex in dLGN, they appear more abundant in vLGN. Differences in retinal terminals are not limited to their anatomy, since retinogeniculate synaptic responses varied significantly in vLGN and dLGN. While retinogeniculate synapses in dLGN exhibited all of the hallmark features of driver inputs, retinal synapses in vLGN elicited weaker postsynaptic responses and displayed features associated with higher levels of convergence on target neurons. These results raise intriguing questions about the different functions of these nuclei in processing light-derived information, as well as differences in the mechanisms that underlie their unique, nuclei-specific development.

## Methods

### Mice

Wild-type C57 mice were obtained from Charles River (Wilmington, MA). *Math5-cre*[66] and *Rosa:lox-stop-lox:tdt* (Jackson Laboratory Stock #007905; referred to here as *Rosa-stop-tdt*) mice were generously provided Dr. C.K. Chen (Baylor College of Medicine, Houston TX). All analyses conformed to National Institutes of Health guidelines and protocols approved by the Virginia Polytechnic Institute and State University Institutional Animal Care and Use Committees.

### Antibodies

Antibodies for the following antigens were purchased: rabbit anti-vesicular glutamate transporter 2 (VGluT2) (Synaptic Systems, Goettingen, Germany; diluted 1:500), rabbit anti-vesicular glutamate transporter 1 (VGluT1) (Synaptic Systems; diluted 1:500), rabbit anti-vesicle associated choline transporter (VAChT)(Synaptic Systems; diluted 1:250), rabbit anti-vesicular GABA transporter (VGAT) (Synaptic Systems; diluted 1:500), rabbit anti-glutamate decarboxylase 65/67 (GAD65) (Millipore Bioscience Research Reagents, Darmstadt, Germany; 1:500), and mouse anti-glutamate decarboxylase 67 (GAD67) (Millipore Bioscience Research Reagents; 1:500). For more details see Table 1. Fluorescently conjugated secondary antibodies were purchased from Invitrogen (diluted 1:1000(Invitrogen/Life Technologies, Grand Island, NY).

**Table 1:** Antibodies used in these studies

**Antigen**	**Isotype**	**Description of immunogen**	**Source/Catalog number**	**References**	**Dilution for immunohistochemistry**
Glutamate Decarboxylase 67 (Gad 67)	Mouse IgG2a	Recombinant Gad67	Millipore #MAB5406	[67-69]	1:500
Glutamate Decarboxylase 65 (Gad 65)	Rabbit IgG	Synthetic peptide containing amino acids 572 to 585 of rat Gad65	Millipore #AB1511	[69,70]	1:500
Vesicular Glutamate Transporter 1 (VGluT1)	Rabbit IgG	Recombinant fusion protein consisting of amino acids 456 to 560 of rat VGlut2	Synaptic Systems #135303	[71,72]	1:500
Vesicular Glutamate Transporter 2 (VGluT2)	Rabbit IgG	Recombinant fusion protein consisting of amino acids 510 to 582 of rat VGlut2	Synaptic systems #135403	[72,73]	1:500
Vesicular GABA Transporter (VGAT)	Rabbit IgG	Synthetic peptide containing amino acids 510 to 525 of rat GABA	Synaptic systems #131103	[74,75]	1:500
Vesicular Acetyl-choline Transporter (VAChT)	Guinea Pig IgG	Fusion protein consisting of amino acids 475 to 530 of rat VAChT	Synaptic systems #139105	[76]	1:250

### Immunohistochemistry

Immunohistochemistry (IHC) was performed on 16-μm coronal cryosectioned tissues as described previously [69]. Briefly, tissue slides were allowed to air dry for 15 minutes before being incubated with blocking buffer (2.5% normal goat serum, 2.5% bovine serum albumin, 0.1% Triton X-100 in PBS) for 30 minutes. Primary antibodies were diluted in blocking buffer and incubated on tissue sections for overnight at 4°C. On the following day, tissue slides were washed in PBS and secondary antibodies diluted 1:1000 in blocking buffer were applied to slides for 1 hour at room temperature. After thoroughly washing in PBS, tissue slides were coverslipped with VectaShield (Vector Laboratories, Burlingame, CA, USA). Images were acquired on a Zeiss LSM 700 confocal microscope. When comparing different ages of tissues or between genotypes, images were acquired with identical parameters. A minimum of three animals (per age) was compared in all IHC experiments. Image analysis was performed in ImageJ using identical parameters for all image sets. Briefly, single color IHC images were binarized and inverted with the ‘Threshold’ tool with NIH ImageJ (*Image > Adjust > Threshold* tool) (http://imagej.nih.gov/ij/). Threshold values for each set of immunofluorescent images (i.e. for each antibody applied) were selected based on the histogram analysis of dLGN images and were then applied to images from both dLGN and vLGN. Threshold values were as follows: VGluT2-IR - *30*; GAD67-IR - *45*; VAChT-IR - *30*. Terminal size analysis was determined with the ‘Analyze Particle’ feature in ImageJ (*Analyze > Analyze Particles* tool) and the fraction of images containing immunoreactivity was determined with the ‘Measure’ feature (*Analyze > Measure* tool). Image analysis was performed on three to five randomly distributed regions of dLGN and vLGN from each hemisphere of at least three wild-type P60 mice. Statistical analysis of quantified images was performed in StatPlus (Analyst Software, Inc.; http://www.analystsoft.com/en/).

### Intraocular injections of anterograde tracers

Intraocular injection of cholera toxin subunit B (CTB) conjugated to Alexa Fluor 488 or Alexa Fluor 594 (Invitrogen/Life Technologies, Grand Island, NY) was performed as described previously [37,49]. Briefly, mice were anesthetized with hypothermia (<P7) or by isoflurane vapors (>P7). The sclera was pierced with a sharp-tipped glass pipette and excess vitreous was drained. Another pipette, filled with a 0.1 to 0.2% solution of CTB, was inserted into the hole made by the first pipette. The pipette containing the CTB was attached to a Picospritzer and a prescribed volume (1 to 3 μl at P3 and 3 to 5 μl for ages > P10) of solution was injected into the eye. After 2 d, mice were euthanized, transcardially perfused with PBS and 4% paraformaldehyde, and brains were post-fixed in 4% paraformaldehyde for 12 hours. Fixed brains were coronally sectioned (80 to 100 μm) on a vibratome (Microm HM 650 V, Thermo Scientific) and mounted in ProLong Gold (Invitrogen/Life Technologies, Grand Island, NY). Retinal projections were analyzed from between three to six animals for each age. Images were acquired on a Zeiss LSM 700 confocal microscope. Terminal size was measured manually in Image J in single optical sections of confocal images from three to five randomly distributed regions of dLGN, IGL, lcvLGN and vLGN from each hemisphere of at least three wild-type mice (per age). Statistical analysis of quantified images was performed in StatPlus (Analyst Software, Inc.; http://www.analystsoft.com/en/).

### Serial block face scanning electron microscopy

Mice were transcardially perfused sequentially with PBS and 4% paraformaldehyde/2% glutaradehyde in 0.1 M cacodylate buffer. Brains were immediately removed, vibratomed (300 μm coronal sections) and vLGN and dLGN were dissected. Tissues were then stained, embedded, sectioned and imaged by Renovo Neural Inc. (Cleveland, OH). Images were acquired at a resolution of 5 nm/pixel and image sets included >200 serial sections (with each section representing 75 nm in the z axis). SBFSEM data sets were 40 μm × 40 μm × approximately 15 μm. Four data sets were analyzed for each region (from a total of three wild-type mice). Data sets were analyzed in TrakEM2 (http://fiji.sc/TrakEM2) [77]. Retinal terminals were identified by the presence of synaptic vesicles and pale mitochondria. Large terminal size was not used as an identifying criteria for retinal terminals. Analysis of data sets was performed independently by two researchers, blind to the tissue of origin for each data set, to ensure unbiased results. Additionally, each analyzed separate sample sets of dLGN and vLGN to ensure terminals were not double counted. Statistical analysis of quantified images was performed in StatPlus (Analyst Software, Inc.; http://www.analystsoft.com/en/).

### Whole-cell patch recordings in visual thalamus

Whole-cell patch recordings were performed as previously described with modifications [78]. P35 wild-type mice were anesthetized with isoflurane, decapitated and brains were rapidly immersed in an ice-cold, oxygenated (95% O_2_/5% CO_2_) solution containing the following (in mM): 26 NaHCO_3_, 234 sucrose, 10 MgCl_2_,11 glucose, 2.5 KCl, 1.25 NaH_2_ PO_4_, 2 CaCl_2_. Coronal sections (300 μm) containing vLGN and dLGN were cut on a vibratome and were incubated in artificial cerebral spinal fluid (ACSF; in mM: 126 NaCl, 2.5 KCl, 1.25 NaH_2_PO_4_, 2.0 MgCl_2_, 26 NaHCO_3_, 2 CaCl_2_, 2 MgCl_2_ and 10 glucose, saturated with 95% O_2_/5% CO_2_, pH 7.3) at 32°C for 25 min and then room temperature. Individual slices were transferred to a recording chamber maintained at 32°C and perfused continuously at a rate of 2.5 ml/min with oxygenated artificial cerebrospinal fluid (ACSF). Patch electrodes were pulled using a two-step puller (Narishige) from borosilicate glass and filled with a solution containing the following (in mM): 117 K-gluconate, 13 KCl,1 MgCl_2_, 0.07 CaCl_2_, 0.01 EGTA, 10 HEPES, 2 Na-ATP, and 0.4 Na-GTP (pH 7.3, 290 osmol/L). The final tip resistance of filled electrodes was 3 to 6 MΩ.

Synaptic responses were evoked by electrical stimulation of the optic tract (OT) using bipolar tungsten electrodes (0.5 MΩ; A-M Systems, Carlsborg, WA) positioned at the caudal ventral border of the LGN [79,80]. Whole-cell recordings were done in voltage-clamp mode at a holding potential of -70 mV and in the presence of SR95531 (10 μM) and the GABA_B_ receptor antagonist 3-minopropyl diethoxymethyl phosphinic acid (10 μM; Tocris Bioscience, Bristol, UK). Single synaptic responses were evoked every 20 s across a range of stimulus intensities (25 to 125 mA). Trains of paired stimuli were delivered (using the stimulus that produced the maximal response) at 20 Hz (50 ms interpulse interval). Whole-cell recordings were obtained using Multiclamp 700 B amplifier (Molecular Devices, Sunnyvale, CA). Data were filtered at 2.5 KHz, digitized at 10 kHz using an interface unit (Digidata 1440A, Molecular Devices), and stored on a computer. Current traces were filtered at 5 kHz; events were detected and amplitudes measured using pClamp 10 software (Molecular Devices).

## Abbreviations

ACSF: artificial cerebrospinal fluid; CTB: cholera toxin subunit B; dLGN: dorsal lateral geniculate nucleus; EPSC: excitatory postsynaptic currents; GAD65: Glutamate decarboxylase 65; GAD67: Glutamate decarboxylase 67; GFP: green fluorescent protein; IGL: intergeniculate nucleus (leaflet); IHC: immunohistochemistry; IR: immunoreactivity; lcvLGN: lateral shell of caudal ventral lateral geniculate nucleus; LGN: lateral geniculate nucleus; OPN: olivary pretectal nucleus; OT: optic tract; PPD: pair-pulse depression; RGCs: retinal ganglion cells; RLP: round synaptic vesicles, large terminal size, and pale-colored mitochondria; RSD: round synaptic vesicles, small terminal size, and dark-colored mitochondria; SBFSEM: serial block face scanning electron microscope; SC: superior colliculus; SCN: the suprachiasmatic nucleus; tdT: tdTomato; VAChT: Vesicular Acetyl-choline Transporter; VGAT: Vesicular GABA Transporter; VGluT1: Vesicular Glutamate Transporter 1; VGluT2: Vesicular Glutamate Transporter 2; vLGN: ventral lateral geniculate nucleus.

## Competing interests

The authors declare that they have no competing interests.

## Authors’ contributions

SH, GC, AM, JSB, and JS carried out the collection, preparation and imaging of tissues, performed IHC, and helped draft portions of the manuscript. GG and WG designed and carried out whole-cell recordings, data collection and helped draft the manuscript. MAF conceived, designed and coordinated the study, and drafted the manuscript. All authors read and approved the final version of the manuscript.
